# Homeostasis or channelopathy? Acquired cell type-specific ion channel changes in temporal lobe epilepsy and their antiepileptic potential

**DOI:** 10.3389/fphys.2015.00168

**Published:** 2015-06-15

**Authors:** Jakob Wolfart, Debora Laker

**Affiliations:** Oscar Langendorff Institute of Physiology, University of RostockRostock, Germany

**Keywords:** hippocampus, homeostasis, kainic acid, pilocarpine, channelacoids

## Abstract

Neurons continuously adapt the expression and functionality of their ion channels. For example, exposed to chronic excitotoxicity, neurons homeostatically downscale their intrinsic excitability. In contrast, the “acquired channelopathy” hypothesis suggests that proepileptic channel characteristics develop during epilepsy. We review cell type-specific channel alterations under different epileptic conditions and discuss the potential of channels that undergo homeostatic adaptations, as targets for antiepileptic drugs (AEDs). Most of the relevant studies have been performed on temporal lobe epilepsy (TLE), a widespread AED-refractory, focal epilepsy. The TLE patients, who undergo epilepsy surgery, frequently display hippocampal sclerosis (HS), which is associated with degeneration of *cornu ammonis* subfield 1 pyramidal cells (CA1 PCs). Although the resected human tissue offers insights, controlled data largely stem from animal models simulating different aspects of TLE and other epilepsies. Most of the cell type-specific information is available for CA1 PCs and dentate gyrus granule cells (DG GCs). Between these two cell types, a dichotomy can be observed: while DG GCs acquire properties decreasing the intrinsic excitability (in TLE models and patients with HS), CA1 PCs develop channel characteristics increasing intrinsic excitability (in TLE models without HS only). However, thorough examination of data on these and other cell types reveals the coexistence of protective and permissive intrinsic plasticity within neurons. These mechanisms appear differentially regulated, depending on the cell type and seizure condition. Interestingly, the same channel molecules that are upregulated in DG GCs during HS-related TLE, appear as promising targets for future AEDs and gene therapies. Hence, GCs provide an example of homeostatic ion channel adaptation which can serve as a primer when designing novel anti-epileptic strategies.

## Introduction

The relationship between epileptic seizures and ion channels is typically focused on the proepileptic (meaning seizure-supporting) nature of ion channel abnormalities. This perspective, embodied in the terms “channelopathy” and “channelepsy” (Hoffman, 1995; Ptacek, 1997; De Lanerolle et al., 2004; Kullmann and Waxman, 2010; D'adamo et al., 2013), is fueled by the increasing number of ion channel mutations discovered in epilepsy patients (Biervert et al., 1998; Charlier et al., 1998; Singh et al., 1998, 2008; Zuberi et al., 1999; Heilstedt et al., 2001; Chioza et al., 2002; Schulte et al., 2006; Cavalleri et al., 2007; Tomlinson et al., 2010; Lachance-Touchette et al., 2011; Weckhuysen et al., 2013), and the seizure phenotypes of corresponding engineered channel mutants (Signorini et al., 1997; Schroeder et al., 1998; Smart et al., 1998; Spigelman et al., 2002; Ludwig et al., 2003; Peters et al., 2005; Huang et al., 2009; Ishii et al., 2009; Riazanski et al., 2011; Hedrich et al., 2014). In contrast to genetic channelopathies, an “acquired channelopathy” is declared when ion channel abnormalities develop independently of the genetic background (Waxman, 2001; Bernard et al., 2004; Poolos and Johnston, 2012). Thus, the prevailing view of progressive acquisition of proepileptic channel properties during epilepsy is conceptually similar to the old “seizures beget seizures” hypothesis (Gower, 1881; Hauser and Lee, 2002; Sills, 2007; Ben-Ari, 2008).

For those studying homeostasis, the acquired channelopathy scenario may appear puzzling; how is it possible that neurons exposed to an environment already hyperexcitable, further enhance their excitability in a potentially self-destructive manner? Usually, biological cells are well equipped to counteract detrimental excitation and especially neurons respond to extrinsic hyperexcitation with intrinsic hypoexcitability on all time scales (Davis and Bezprozvanny, 2001; Turrigiano and Nelson, 2004; Marder and Goaillard, 2006; Meier et al., 2015). Are these mechanisms of protective homeostasis disabled in epilepsy? And if so, is it the rule or rather the exception? It is important to answer these questions, because it will help to understand the mechanisms of seizures and antiepileptic drugs (AEDs). The prevailing AED strategy is to inhibit excitatory channels such as sodium (Na) and calcium (Ca) channels (Löscher et al., 2013). A more recent approach is to support a specific potassium (K) channel (further discussion below) (Tatulian et al., 2001; Faulkner and Burke, 2013). Generally, AED strategies imply relatively fixed ion channel repertoires, but channels are dynamically adjusted all the time and these changes are not well understood in particular with respect to epilepsy.

Most of the discussion on channel-related AED mechanisms of action takes place without mentioning cell types even though AED channel targets can have behaviorally opposed effects depending on which cell types are affected (Prakriya and Mennerick, 2000; He et al., 2002). Hence, to discuss antiepileptic strategies in a meaningful manner, the first step is to obtain an overview on available information about channel molecule alterations, cell types, and epilepsy model methods. The present review focuses on temporal lobe epilepsy (TLE), because the majority of the available data concern this most common form of partial epilepsies, which in turn account for 60% of all adult epilepsy cases (Tellez-Zenteno and Hernandez-Ronquillo, 2012). Some of the discussed channel mechanisms might also be relevant for other epilepsies as many TLE animal models display generalized seizures. Studies on the principal neurons of the hippocampal formation, especially *cornu ammonis* 1 (CA1) and dentate gyrus (DG), outnumber studies on other areas by far. This fact is mirrored in our list of acquired ion channel alterations (Table 1). Other cell types are expected to gain importance in the future and are also discussed in respective sections. We especially highlight channels with net inhibitory effects and relate these to existing or promising AED mechanisms of action (Table 2), asking whether certain cell types can signpost effective molecule target combinations for future AED and antiepileptic gene therapies.

**Table 1:** **Ion channel changes during temporal lobe epilepsy**.

**Type of cells and TLE/model**	**Ion channel/current**	**Regulation/methods**	**References**
**DG GRANULE CELLS**			
HS/noHS human	I_Kir2.x_	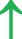 funct (vc, 0.1 mM Ba^2+^)	Stegen et al., 2009
HS/no HS human	K_ir_2.2	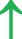 prot IP	Stegen et al., 2012
HS/noHS human	GABA_A_α1/α2 GABA_A_α3 GABA_A_β2/β3 GABA_A_γ2	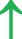 prot IP ± prot IP 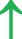 prot IP 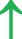 prot IP	Loup et al., 2000
HS/noHS human	GABA_B_R1a-b	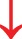 prot IP	Munoz et al., 2002
HS/noHS human	HCN1	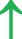 RNA_ISH_, prot IP	Bender et al., 2003
HS/noHS human	I_H_ HCN1	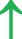 funct (vc, ZD7288) 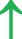 prot IP	Stegen et al., 2012
TLE/autopsy human	K_v_7.5	± prot IP	Yus-Najera et al., 2003
TLE/autopsy human	Na_v_1.1/1.2/1.3	± RNA_ISH_	Whitaker et al., 2001
TLE/autopsy human	Ca_v_1.2 Ca_v_1.3 Ca_v_2.2/2.3 Ca_v_2.1	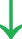 prot IP ± prot IP ± prot IP 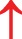 prot IP	Djamshidian et al., 2002
HS-iKA mouse	I_Kir2.x_ K_ir_2.1/2.2/2.3/2.4	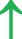 funct (vc, 40μM Ba^2+^) 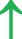 prot IP	Young et al., 2009
HS-iKA mouse	K_2P_1.1 K_2P_6.1	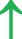 prot IP ↑ prot IP	Young et al., 2009
HS-iKA mouse	I_Kv1.x_ K_v_1.1 K_ir_2.1	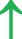 funct (vc, DTX) 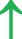 RNA_SC-PCR_, prot IF 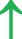 RNA_SC-PCR_	Kirchheim et al., 2013
HS-iKA mouse	GABA_A_ α1/α5 GABA_A_ α2 GABA_A_ α3 GABA_A_ γ2	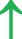 prot IP 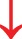 prot IP ± prot IP 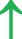 prot IP	Bouilleret et al., 2000
HS-iKA mouse	GABA_A_α2	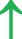 prot IF	Knuesel et al., 2001
HS-iKA mouse	I_tGABA-A_	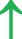 funct	Young et al., 2009
hKindl rat	GABA_A_α4	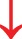 _syn_ 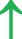 _ex_ prot EM	Sun et al., 2007
hKindl rat	I_Na_	± funct	Ketelaars et al., 2001
aKindl rat	Na_v_1.1/1.2/1.6 Na_v_1.6	± prot IF ± RNA_ISH_	Blumenfeld et al., 2009
aKindl mouse	Na_v_1.6	± prot IF	Blumenfeld et al., 2009
sPilo rat	K_v_1.4 K_v_4.2/KChiP2	± prot IP ± prot IP	Monaghan et al., 2008
sPilo rat	I_GABAeff_ I_GABApot_	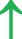 funct ± funct	Gibbs et al., 1997
sPilo rat	I_GABAeff_ I_GABApot_ GABA_A_α1 GABA_A_α2/α3 GABA_A_α4 GABA_A_β1 GABA_A_β2 GABA_A_β3 /δ /ε GABAγ1/γ2/γ3	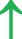 funct ± funct 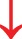 RNA_SC-PCR_ ±RNA_SC-PCR_ 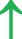 RNA_SC-PCR_ 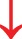 RNA_SC-PCR_ ± RNA_SC-PCR_ 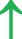 RNA_SC-PCR_ ± RNA_SC-PCR_	Brooks-Kayal et al., 1998
sPilo mouse	GABA_a_α4 GABA_a_δ GABA_a_γ2	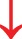 _1–4days_ 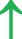 _30days_ prot IP 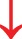 prot IP ±_1–4days_ 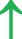 _60days_ prot IP	Peng et al., 2004
sPilo rat	GABA_A_β2 GABA_A_β3 GABA_A_δ GABA_A_γ2	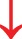 _surface_ prot W 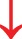 _surface_ prot W ± prot_surface_ ( 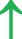 ), funct 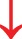 _surface_ prot W	Goodkin et al., 2008
sPilo rat	HCN1	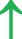 RNA_ISH_	Bender et al., 2003
sPilo rat	TRP3 TRP6	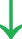 _1days–1week_ prot IP 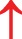 _1days–1week_ prot IP	Kim et al., 2013
sPilo rat, mouse	I_Cav_ Ca_v_3.2	± funct ± prot IF	Becker et al., 2008
sPilo mouse	I_tGABA-A_ I_pGABA-A_ GABA_A_α GABA_A_δ GABA_A_γ2	± funct 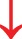 funct 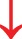 _syn/ex_ prot EM 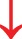 _syn/ex_ port EM 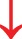 syn 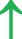 _ex_ prot EM	Zhang et al., 2007
P20 sPilo rat	GABA_A_α1	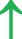 RNA_SC-PCR_	Raol et al., 2006b
P20 sKA rat	GABA_A_α1	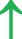 RNA_SC-PCR_	Raol et al., 2006b
sKA rat	K_v_4.2	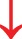 _3h,6h_ 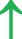 _24h_ RNA_ISH_	Francis et al., 1997
sKA rat	Na_v_1.1 Na_v_1.2 Na_v_1.3	± _3–24h_ RNA_ISH_ 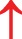 _3–6h_ ± _24h_ RNA_ISH_ 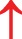 _3–6h_ 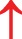 _24h_ RNA_ISH_	Bartolomei et al., 1997
**DG INTERNEURONS**			
sPilo rat	KChiP1 (hilus)	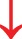 prot	Monaghan et al., 2008
sPilo rat	tGABA_A_(BCs) GABA_A_δ(BCs)	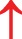 _6–8days_ funct 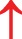 _1week_ prot IF	Yu et al., 2013
sPilo mouse	GABA_A_δ(BCs)	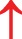 prot IP	Peng et al., 2004
**DG GLIAL CELLS**			
HS/noHS human	I_Kir_	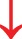 funct	Hinterkeuser et al., 2000
HS/noHS human	K_ir_4.1 (hilus)	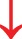 prot	Heuser et al., 2012
sPilo rat	K_2P_3.1	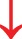 prot	Kim et al., 2008b
**CA1 PYRAMIDAL CELLS**			
TLE/autopsy human	K_v_7.5	± prot IP	Yus-Najera et al., 2003
TLE/autopsy human	Na_v_1.1/1.3 Na_v_1.2	± RNA_ISH_ 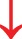 RNA_ISH_	Whitaker et al., 2001
TLE/autopsy human	Ca_v_1.2/1.3 Ca_v_2.1/2.2/2.3	± prot IP ± prot IP	Djamshidian et al., 2002
HS-iKA mouse	GABA_A_α2	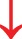 prot IF	Knuesel et al., 2001
HS-iKA mouse	GABA_A_α1/α3/α5 GABA_A_α2 GABA_A_γ2	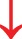 prot IP ± prot IP 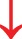 prot IP	Bouilleret et al., 2000
hKindl rat	I_Na-window_	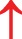 funct	Ketelaars et al., 2001
aKindl rat	Na_v_1.1/1.2/1.6 Na_v_1.6	± prot IF ± RNA_ISH_	Blumenfeld et al., 2009
aKindl mouse	Na_v_1.6	± prot IF	Blumenfeld et al., 2009
sPilo rat	I_A-type/dend_	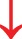 funct (cc, 5 mM 4-AP)	Bernard et al., 2004
sPilo rat	K_v_4.2/KChiP2	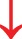 prot IP	Monaghan et al., 2008
sPilo rat	I_SK_	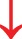 funct (vc, UCL1684)	Schulz et al., 2012
sPilo rat	I_GABAeff_ I_GABApot_	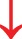 funct 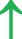 funct	Gibbs et al., 1997
sPilo rat	I_Cl_ CLC2	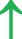 funct 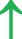 prot IF, W	Ge et al., 2011
sPilo rat	I_H_/_dend_ I_H/soma_	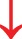 _1h–5weeks_ funct ± _3days–5weeks_ funct	Jung et al., 2007, 2011
sPilo rat	I_H_ HCN2	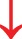 funct (vc, ZD7288) 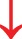 prot (surface)	Marcelin et al., 2009
sPilo rat	I_Cav_	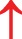 funct (cc, 1 mM Ni^2+^)	Sanabria et al., 2001
sPilo rat	TRP3 TRP6	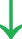 _1days–1week_ prot IP 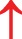 _1days–1week_ prot IP	Kim et al., 2013
sPilo rat	I_Cav3_	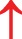 funct (vc, 0.1 mM Ni^2+^)	Su et al., 2002
sKA rat	K_v_4.2	± _3h, 6h, 24h_ RNA_ISH_	Francis et al., 1997
sKA rat	I_Kv1.x_ K_v_1.1	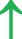 _14days_ 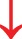 _30days_ funct (cc, 50 μM 4-AP) 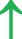 _14days_ 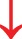 _30days_ prot IF/W	Sosanya et al., 2014
sKA rat	HCN1 HCN2	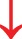 RNA_ISH_ 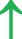 RNA_ISH_	Brewster et al., 2002
sKA rat	I_H_	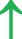 _1–2days_ 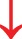 _28–30days_ funct	Shin et al., 2008
sKA rat	Na_v_1.1 Na_v_1.2 Na_v_1.3	± _3–24h_ RNA_ISH_ ± _3–24h_ RNA_ISH_ ± _3h_ 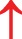 _6h_± _24h_ RNA_ISH_	Bartolomei et al., 1997
**CA1 GLIAL CELLS**			
TLE/autopsy human	K_ir_4.1	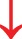 prot IP	Heuser et al., 2012
sPilo rat	K_2P_3.1	± prot	Kim et al., 2008b
sKA rat	K_v_1.3	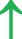 funct	Menteyne et al., 2009
**CA2 PYRAMIDAL CELLS**			
HS/noHS human	GABA_A_α2 GABA_A_α1/α3	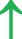 prot IP 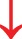 prot IP	Loup et al., 2000
TLE/autopsy human	Na_v_1.1/1.3 Na_v_1.2	± RNA_ISH_ 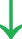 RNA_ISH_	Whitaker et al., 2001
TLE/autopsy human	Ca_v_1.2/1.3 Ca_v_2.1/2.2/2.3	± prot IP ± prot IP	Djamshidian et al., 2002
noHS-aKindl rat	Na_v_1.1/1.2/1.6 Na_v_1.6	± prot IF ± RNA_ISH_	Blumenfeld et al., 2009
**CA3 PYRAMIDAL CELLS**			
HS/noHS human	Na_v_1.1/1.3 Na_v_1.2	± RNA_ISH_ 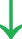 RNA_ISH_	Whitaker et al., 2001
TLE/autopsy human	Ca_v_1.3, Ca_v_2.2/2.3 Ca_v_2.1 Ca_v_1.2	± prot IP ± prot IP 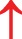 prot IP 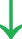 prot IP	Djamshidian et al., 2002
HS-iKA mouse	GABA_A_α1/α2 GABA_A_ α5 GABA_A_γ2	± prot IP 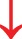 prot IP 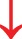 prot IP	Bouilleret et al., 2000
aKindl rat	Na_v_1.1/1.2 Na_v_1.6	± prot IF 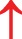 prot IF, RNA_ISH_	Blumenfeld et al., 2009
aKindl mouse	I_persist inward_ Na_v_1.6	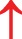 funct 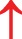 prot IF, RNA_ISH_	Blumenfeld et al., 2009
sKA rat	K_v_4.2	± _3h,6h_ 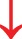 _24h_ RNA_ISH_	Francis et al., 1997
sKA rat	I_Kv1.x_ K_v_1.1	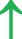 _14days_ 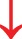 _30days_ funct 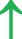 _14days_ 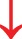 _30days_ prot IF/W	Sosanya et al., 2014
sKA rat	HCN1 HCN2	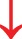 RNA_ISH_ 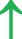 RNA_ISH_	Brewster et al., 2002
sKA rat	Na_v_1.1 Na_v_1.2 Na_v_1.3	± _3–24h_ RNA_ISH_ ± _3–24h_ RNA_ISH_ ± _3h_ 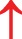 _6h_± _24h_ RNA_ISH_	Bartolomei et al., 1997
sPilo rat	TRP3 TRP6	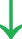 _1days–1week_ prot IP 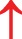 _1days–1week_ prot IP	Kim et al., 2013
**CA INTERNEURONS**			
ventral of iKA	I_H_(CA3 O-LM)	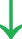 funct	Dugladze et al., 2007
sPilo rat	KChiP1 (CA3 SP)	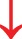 prot IP	Monaghan et al., 2008
**SUBICULAR NEURONS**			
sPilo rat	I_Ca_ (PCs)	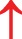 funct (cc, 1 mM Ni^2+^)	Wellmer et al., 2002
**AMYGDALA NEURONS**			
a/hKindl rat	K_v_7.2	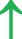 prot IP	Penschuck et al., 2005
**CORTICAL NEURONS**			
hKindl rat EC	I_Nav_ (layer II stellate cells) Na_v_1.2/6 (layer II stellate cells) Na_v_1.1/3 (layer II stellate cells)	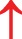 funct 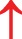 prot IF ± prot IF	Hargus et al., 2013
hKindl rat PC	K_v_1.6 (interneurons)	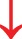 funct, prot IF	Gavrilovici et al., 2012
sKA rat EC	I_H_ (layer III PCs)	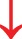 _24h_ 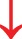 _1week_ funct (dendritic)	Shah et al., 2004
**THALAMIC NEURONS**			
sPilo mouse	I_A_ (VM relay cells) K_v_4.2 (surf)	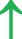 funct 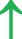 prot IF	Smith et al., 2012
sPilo mouse	I_T-Type_ (relay cells) Ca_v_3.1 (thalamus) Ca_v_3.2 (thalamus) Ca_v_ 3.3 (thalamus)	± _4h_ 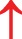 _10days_ 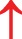 _31days_ funct ± _4h/10days/31days_ RNA_PCR_ ± _4h_ 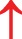 _10days_ 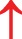 _31days_ RNA_PCR_ ± _4h_ 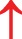 _10days_ ± _31days_ RNA_PCR_	Graef et al., 2009
**TISSUE LEVEL ANALYSIS**			
HS/noHS human	K_v_4.2 (hipp)	↑ prot W	Aronica et al., 2009
TLE/autopsy human	K_v_7.5 (temporal cortex)	± prot IP	Yus-Najera et al., 2003
TLE/autopsy human	CLC2 (temporal lobe)	↓ RNA_PCR_	Bertelli et al., 2007
HS-iKA mouse	K_ir_3.2 (DG ML)	↓ prot IP	Young et al., 2009
sKA rat	HCN1/2 (EC)	↓ _24h_ = _1week_ prot W	Shah et al., 2004
sKA rat	HCN1 (CA1) HCN2 (CA1)	↑ _1–2_↓ _28–30days_ prot W ± _1–2days_ ↓ _28–30days_ prot W	Shin et al., 2008
sKA rat	HCN1 (CA1/DG) HCN1 (CA3) HCN1 (EC) HCN2 (CA1) HCN2 (CA3) HCN2 (DG) HCN2 (EC)	± _24h_↓ _7days/6weeks_ RNA_PCR_ ± _24h_↓ _7days_ ± _6weeks_ RNA_PCR_ ± _24h/7days/6weeks_ RNA_PCR_ ↓ _24h/7days/6weeks_ RNA_PCR_ ↓ _24h_± _7days/6weeks_ RNA_PCR_ ↓ _24h_± _7days_ ↓ _6weeks_ RNA_PCR_ ↓ _24h_± _7days/6weeks_ RNA_PCR_	Powell et al., 2008
sPilo rat	K_v_4.2 (CA1)	↓ _30days_ RNA_PCR_	Bernard et al., 2004
sPilo rat	K_v_1.4/3.3/3.4/4.2/4.3 (DG)	± RNA_PCR_	Rüschenschmidt et al., 2006
sPilo rat	KChiP2 (CA1) KChiP2 (DG) K_v_4.2 (CA1) K_v_4.2 (DG)	↓ _1week_ ↓ _4weeks_ prot IF ↓ _1week_ ± _4weeks_ prot IF ↓ _1week_ ± _4weeks_ prot IF ↓ _1week_ ± _4weeks_ prot IF	Monaghan et al., 2008
sPilo rat	K_v_4.2 (CA1/2) K_v_4.2 (CA3) K_v_4.2 (DG)	↑ _2days_ ± _50days_ prot IF ↑ _2days_ ↓ _50days_ prot IF ± prot IF	Su et al., 2008
sPilo rat	K_2P_5.1 (CA1) K_2P_5.1 (CA3) K_2P_5.1 (DG)	↓ _3days–5weeks_ prot IF (cell loss) ↑ _3days–5weeks_ prot IF ↑ _3days–5weeks_ prot IF	Kim et al., 2009
sPilo rat	SK1/2 (hipp) SK3 (hipp) SK1 (hipp) SK2/3 (hipp)	↓ _10days_ ± _chronic_ prot W ↓ prot W ± RNA_PCR_ ↓ RNA_PCR_	Oliveira et al., 2010
sPilo rat	SK1/2/3 (CA1) SK2 (CA1) SK1/3 (CA1)	↓ RNA_PCR_ ↓ prot W ± prot W	Schulz et al., 2012
sPilo rat	BK (hilus/CA3)	↓ prot IF/W	Pacheco Otalora et al., 2008
sPilo rat	HCN1 (CA1) HCN2 (CA1)	↓ _3,6days_ ↓ _3–5weeks_ prot W ↓ _3,6days_ ± _3–5weeks_ prot W	Jung et al., 2007
sPilo rat	HCN1 (CA1)	↓ RNA_PCR_	Marcelin et al., 2009
sPilo rat	HCN1 (CA1-3/DG) HCN2 (CA1) HCN2 (CA2/3) HCN2 (DG) HCN4 (CA1-3/DG)	↑ _12h_ ± _2weeks_ ↑ _11weeks_ prot IP ↓ _12h_ ± _7days_ ↓ _5weeks_ prot IP ± _12h–5weeks_ prot IP ↓ _12h–5weeks_ prot IP ± _12h–5weeks_ prot IP	Oh et al., 2012
sPilo rat	K_ir_2.1 (amyg, hipp, ctx) K_ir_4.1 (hipp) K_ir_4.1 (amyg, striat, ctx) K_ir_5.1 (amyg, hipp, ctx)	± prot W ± prot W IP ↑ prot W IP ± prot W	Nagao et al., 2013
sPilo rat /mouse	Ca_v_3.2 (CA1)	↑ _5days_ ± _chronic_ RNA_PCR_, prot W	Becker et al., 2008
sPilo mouse	Ca_v_3.1 (hippocampus) Ca_v_3.2 (hippocampus) Ca_v_3.3 (hippocampus)	± _10days/31days_ RNA_PCR_ ± _10days_ ↓ _31days_ RNA_PCR_ ± _10days/31days_ RNA_PCR_	Graef et al., 2009
noHS-aKindl rat	HCN1 (CA1/3/DG/EC) HCN2 (CA1/DG/EC) HCN2 (CA3)	± _partial/full_ RNA_PCR_ ± _partial/full_ RNA_PCR_ ± _partial_ ↓ _full_ RNA_PCR_	Powell et al., 2008
vKA rat	Ca_v_1.2/1.3 (CA1-3/DG) Ca_v_2.1/2.2 (CA1-3/DG) Ca_v_ α2 (CA3 astrocyt)	± prot IP ± prot IP ↑ prot IP	Westenbroek et al., 1998

*1st column: aKindl, hKindl, Epilepsy susceptibility model via repeated electrical stimulation (kindling) of amygdala or hippocampal areas; BCs, Basket cell interneurons; CLC, Chloride channel; dend, Dendritic recording; EC, Entorhinal cortex; HS/noHS human, Tissue from TLE patients with hippocampal sclerosis (HS) compared to samples from TLE patients with mild or no HS (noHS); HS-iKA, intrahippocampal unilateral kainic acid (KA) injection status epilepticus (SE) TLE animal model with severe HS; KChiP, K channel interacting protein (enhances K_v_4 function); O-LM, Interneurons projecting from stratum oriens to stratum lacunosum moleculare; PC, Piriform cortex sKA, systemic (intraperitoneal) KA injection SE TLE model; sPilo, systemic pilocarpine injection SE TLE model using adult rats; SP, Interneurons scattered in stratum pyramidale; ventral of iKA, Same iKA model as above but data collected in ventral (noHS) areas; vKA, intraventricular KA injection SE TLE model shows sclerosis in CA3. 2nd column: For ion channel nomenclature see text; amyg, Amygdala; ctx, Cortex; hipp, Hippocampus; I_X_, Current x measured (t/pGABA_A_, tonic/phasic current via GABA_A_Rs; GABA_eff/pot_, efficacy or potency of GABA application; striat, Striatum TRP, Transient receptor potential cation channels; 3rd column: ↑/↓, Up-/downregulation (interpreted as antiepileptic/stabilizing (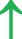

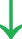
) or as proepileptic/excitatory (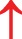

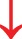
) effect; ±, No difference; subscripts, time period in which the changes took place (otherwise chronic) and/or method (full/partial refers to kindling procedure) and/or subcellular location like synaptic (syn), extra - and/or peri synaptic (ex), and surface (surf); funct, Functionally (electrophysiologically) verified via, voltage-clamp (vc) or current-clamp (cc), or with pharmacology (drug); prot, Protein detection via immunofluorescence (IF) or immunoperoxidase (IP) or western blot (W) labeling, or electron microscopy (EM); ISH, RNA detection via in situ hybridization; P10/20 sPilo, systemic pilocarpine injection SE TLE model using postnatal day 10 or 20 rats; PCR, mRNA detection via reverse transcriptase (RT)PCR on the tissue level; SC-PCR, Single cell (cell type specific) RT-PCR*.

**Table 2:** **Inhibitory ion channel changes with antiepileptic potential**.

**Ion channel**	**AED, Seizure model**	**Cell type/Method**	**References**
**POTASSIUM CHANNELS**			
K_2P_2.1 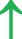	GenTher (hip, EC, pre SE)/sPilo	Hip neur recs, CellCult, EEG, behavior	Dey et al., 2014
K_ir_1.1 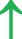	Pregabaline/A	Thal neur recs, HetEx	Lee and Liou, 2014
K_ir_2.3 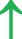	Tenidap/A*	HetEx recs	Liu et al., 2002
K_ir_2.3 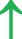	Tenidap/A*/sPilo	EEG, behavior	Xu et al., 2013a
K_ir_3.1 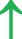	ML297/MES/PTZ	HetEx recs, behavior	Kaufmann et al., 2013
K_ir_3.4 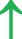		
K_ir_6.2 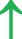	Pregabaline/A H19-7	CellCult hip neur recs	Huang et al., 2006
K_ir_6.x 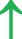	Cromakalim/diazoxide anoxia seizure model	Field recs	Mattia et al., 1994
K_v_1.1 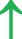	PTZ	Behavior	Lu et al., 2008
K_v_1.1 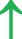	GenTher (neo, post SE)/NCTX iTX	NCTX PC recs, EEG	Wykes et al., 2012
K_v_1.1 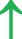	Rapamycin/sKA	Behavior	Sosanya et al., 2014
K_v_7.2/7.3 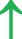	ICA-27243/MES/PTZ/aKindl	Behavior, HetEx recs, Hip neur recs	Roeloffs et al., 2008; Wickenden et al., 2008
K_v_7.x? 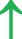	Somatostatin/Gen sPent/sKA	CA1 PCs, field recs, behavior	Qiu et al., 2008
K_v_7.2/7.3 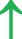	Flupirtine/A*/sKA/FNeo	EEG, behavior	Raol et al., 2009
K_v_7.2/7.3 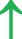	Retigabine/A	CA3 PCs recs	Kim et al., 2012
K_v_7.2/7.3 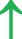	Retigabine/A/case	EEG, behavior	Walleigh et al., 2013
K_v_7.2/7.3 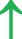	Retigabine/A	HetEx recs	Schenzer et al., 2005; Zhou et al., 2013
K_v_7.2–7.5 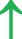	ICA-105665 i.p./P_2_	EEG, behavior	Kasteleijn-Nolst Trenite et al., 2013
K_v_ Del 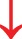	Lamotrigine/A	CA1 PC recs	Grunze et al., 1998
K_v_ Del 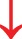	Levetiracetam/A	Hip neur recs, CellCult	Madeja et al., 2003
K_v_ A-type 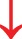	Lamotrigine/A	Hip neur recs, CellCult	Huang et al., 2004
hERG 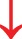	Lamotrigine/A Phenytoin/A	HetEx recs	Danielsson et al., 2003, 2005
KCa (SK) 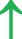	EBIO/MES, PTZ	Behavior	Anderson et al., 2006
KCa (BK) 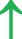	Zonisamide/A H19-7	Hip neur recs, CellCult	Huang et al., 2007
KCa2 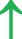	SKA-19 i.p./aKindl/*in vitro* PTX/4-AP	CA1 PC recs	Coleman et al., 2015
Na_v_ 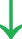			
**GABA_A_ RECEPTORS AND OTHER CHLORIDE CHANNELS**			
GABA_Aα1_ 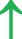	GenTher (DG, pre SE)/sPilo	DG, EG, behavior	Raol et al., 2006a
I_GABAA_ 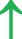	Retigabine/A	Cort neur recs, CellCult	Otto et al., 2002
Cl NpHR 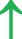	Optogenetic act/NCTX iTX	EEG	Wykes et al., 2012
GABA_Aδ_ 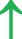	GenTher/CTZ/sKA	CA1 PC recs, behavior	Sun et al., 2013
**HCN CHANNELS**			
I_H_ 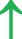	Lamotrigine/A	CA1 PCs, dendritic recs	Poolos et al., 2002
I_H_ 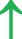	Lamotrigine/A	Interneurons (CA1_O-LM?_)	Peng et al., 2010
I_H_ 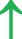	Gabapentin/A	CA1 PCs	Surges et al., 2003

1st column: ↑ ↓, Manipulated up (↑) and down (↓) regulation of respective ion channel interpreted as pro- or anticonvulsive (red and green, respectively). 2nd column: /A, approved drug as AED or /A^*^ for another indication; /case, case report of centroparietal focal seizures stopped by Retigabine; /P_*2*_, phase 2 clinical study; /GenTher, in vivo gene therapy via stereotaxic intracranial injection of viral vectors in hippocampus (hip), entorhinal cortex (EC) or neocortex (neo). Tested in animal models: /aKindl, amygdala kindling seizure model; /FNeo, rat model of neonatal seizures via convulsant gas flurothyl; MES, maximal electroshock model of epilepsy; NCTX iTX, motorcortex tetanus toxin injection model of neocortical epilepsy; PTZ, systemic pentylenetetrazol injection model of epilepsy; hKindl, hippocampal kindling to SE seizure model; iKA, intrahippocampal unilateral kainic acid injection SE TLE model; Neo sKA, systemic KA injection SE model of neonatal seizures; sPilo, systemic pilocarpine injection SE TLE model; 3rd column: CellCult, cell culture, e.g., of primary fetal cortical neurons; Cor neur, cortical neurons; field recs, extracellular field recordings; HetEx, heterologous expression and pharmacological testing of cloned channels; Hip neur, hippocampal neurons, Cl NpHR, chloride pump halorhodopsin from Natronomonas pharaonis, NpHR; recs, electrophysiological recordings; Thal neur, thalamic neurons.

## Temporal lobe epilepsy

TLE seizures start in the temporal lobe and impair the consciousness, among other symptoms (Blumenfeld and Meador, 2014). TLE is often refractory to AEDs; therefore, anterior temporal lobe resection is a standard treatment (De Tisi et al., 2011). In its most common form, TLE affects the medial temporal/limbic network with involvement of the entorhinal cortex, the amygdala, and the hippocampus with the DG and the CA regions 1-3 (hippocampus *sensu strictu*) (Spencer, 2002). The neuropathological correlate of TLE is hippocampal sclerosis (HS) (Margerison and Corsellis, 1966; Curia et al., 2014) and quite often is HS interpreted to be the cause of TLE (Blümcke et al., 2012). However, as human TLE is heterogeneous, it appears difficult to find even simple correlations between the severity of HS and epileptic seizures, let alone the certainty that HS causes TLE or indeed vice versa (King et al., 1997; Jefferys, 1999; Blümcke et al., 2002; Mathern et al., 2002; Sutula et al., 2003; De Lanerolle and Lee, 2005; Briellmann et al., 2007; Mueller et al., 2007). A widespread criterion for HS diagnosis is loss of more than half of the CA1 PCs (Wyler et al., 1992). Other structural changes associated to HS, are sprouting of the mossy fiber axons of DG granule cells (GCs) (Sutula et al., 1989), changed GC morphology (Isokawa and Levesque, 1991), and GC dispersion (GCD) (Houser, 1990). Severe HS is correlated with severe GCD (Thom et al., 2002), although this relationship is not always strict (Blümcke et al., 2013). Occasionally, studies counting cells per area confuse GCD with degeneration of GCs. However, GCs are only lost in extreme HS (Wyler grade IV), i.e., when most of the CA1 PCs have already degenerated (Wyler et al., 1992). More than 90% of the hippocampi resected during epilepsy surgery exhibit HS (Blümcke et al., 2012) but it should be kept in mind that the decision on surgery itself depends on HS because the success rates of epilepsy surgery are higher with diagnosis of a lesion (Jobst and Cascino, 2015). Unbiased postmortem studies reveal HS in about half of TLE patients (Thom et al., 2010).

Epilepsy surgery offers direct experimental access to living human hippocampal tissue but lack of proper control tissue makes animal models indispensable. Different approaches were used to simulate a chronic epileptic state with anatomical changes resembling TLE. Since human TLE is heterogeneous and has unknown causes, animal models can only reproduce partial aspects of the disease (Coulter et al., 2002; Morimoto et al., 2004). One hypothesis for TLE is an initial precipitating injury (Mathern et al., 2002). Therefore, most animal models generate a chronic epileptic state via one status epilepticus (SE). Widespread is SE induction via i.p. (systemic) injection of kainate (sKA) (Nadler et al., 1978; Ben-Ari, 1985) or pilocarpine (sPilo) (Turski et al., 1983, 1989). The sKA and sPilo models produce generalized seizures and bilateral brain damage, the extent of which depends on the SE-termination protocol (Schwob et al., 1980; Turski et al., 1983). Often, no HS or only mild forms of it (noHS) occur in sKA and sPilo models (Okazaki et al., 1999; Scharfman et al., 2000; Dietrich et al., 2005; Curia et al., 2014). In contrast, development of HS and GCD, as well as focal spontaneous TLE seizures, can be induced by intracranial (intrahippocampal) kainate injection (iKA) (Suzuki et al., 1995; Fritschy, 2004). Another TLE model, which reproduces HS and chronic (bilateral) seizures, is perforant path stimulation-induced non-convulsive- (Kienzler et al., 2009) or convulsive SE (Bumanglag and Sloviter, 2008). In addition, there are different forms of electrical stimulation (“kindling”) of amygdala or hippocampus (aKindl, hKindl) which mostly evoke seizures only during the stimulation (i.e., no chronic epilepsy), (Goddard et al., 1969; Mcnamara, 1984; Morimoto et al., 2004).

## Dentate gyrus

The DG is often viewed as a strategic “gate keeper” of the hippocampus and failure in its filter function was hypothesized to be a potential cause for TLE seizures (Heinemann et al., 1992; Lothman et al., 1992; Hsu, 2007; Krook-Magnuson et al., 2015). An alternative hypothesis is that the DG does not actively contribute to hippocampal seizures (Sloviter, 1994; Liu et al., 2000; Harvey and Sloviter, 2005). The following changes were considered responsible for a proepileptic role of the DG (De Lanerolle et al., 1992; Mody et al., 1992b): (i) mossy fiber sprouting (Tauck and Nadler, 1985; Sutula et al., 1989), (ii) loss of specific interneurons (Sloviter, 1987; Magloczky and Freund, 2005), and (iii) intrinsic hyperexcitability of the principal DG neurons, the GCs (Magloczky et al., 1997; Beck et al., 1998; Dietrich et al., 1999; Coulter, 2000; De Lanerolle et al., 2003; Selke et al., 2006; Mehranfard et al., 2014a). In contrast to the last point, many studies concluded that TLE does not change DG GCs intrinsically (Mody et al., 1992a; Beck et al., 1996; Isokawa, 1996; Molnar and Nadler, 1999; Okazaki et al., 1999; Scharfman et al., 2003; Dietrich et al., 2005; Beck and Yaari, 2008). Contrary to both prior hypotheses, we found a *decrease* of the intrinsic excitability of GCs which was due to a reduction in input resistance (R_in_); it occurred in samples of TLE patients with HS vs. mild/no HS as well as in iKA vs. control mice (Stegen et al., 2009, 2012; Young et al., 2009; Kirchheim et al., 2013). A reasonable question is: why are there so many disparate results on the same cell type (Vida, 2009)? With rare exceptions (Isokawa and Mello, 1991; Mehranfard et al., 2014b), most studies reporting unchanged GCs were those employing TLE models without HS. In TLE patients and the iKA TLE model, the R_in_ of DG GCs correlates with the degree of HS (Stegen et al., 2009, 2012; Young et al., 2009). Although it cannot be ruled out that some of the studies missed R_in_ differences due to methodological procedures, such as applying minimum R_in_ as cell selection criterion, low seal resistance sharp electrodes, or by dissociating GC somata from their dendritic conductances (Mehranfard et al., 2014b), the conservative conclusion currently is: in TLE with HS, the ion channel expression of GCs is more drastically changed than in TLE without HS. It is important to note that GC channel adaptations only occur in the HS area, i.e., where GCD and neurodegeneration are clearly visible, but not outside of this HS focus. Ventral parts of the ipsilateral DG of iKA mice as well as the contralateral DG may even harbor hyperexcitable GCs (Le Duigou et al., 2008; Young et al., 2009; Häussler et al., 2012).

The molecular mechanism behind the reduced excitability of GCs is mainly transcriptional upregulation of K leak channels, i.e., channels that are open at resting membrane potential (V_rest_) (Stegen et al., 2009, 2012; Young et al., 2009). Specifically, these are inwardly rectifying K (K_ir_) channels of classic leak subtype K_ir_2.1-4 and two pore domain K leak channels of subtype K_2P_1.1 and K_2P_6.1. In addition, elevated tonic chloride (Cl) leak conductances mediated by gamma amino butyric receptors type A (GABA_A_Rs) were detected in GCs of iKA mice (Young et al., 2009). Such tonic GABA_A_ currents are likely mediated by extrasynaptic GABA_A_Rs composed of α4-6 plus β, and γ2 or δ subunits (Peng et al., 2002; Zhang et al., 2007; Glykys et al., 2008) which could underlie epilepsy-related changes (Peng et al., 2004). In the adult stage, the Cl equilibrium potential (E_Cl_) of GCs is between V_rest_ and action potential (AP) threshold, although E_Cl_ can change during TLE (Palma et al., 2006; Huberfeld et al., 2007; Pathak et al., 2007; Khirug et al., 2010; Barmashenko et al., 2011). Therefore, the functional influence of TLE-related GABA_A_ leak elevation is an enhancement of shunting inhibition. To explain the latter: if a large conductance (1/R) is added, its influence via Ohm's law (U = R × I) will minimize the voltage impact (U) of any further input currents (I). All conductances have this influence, but the counterintuitive effect of “inhibitory depolarization” occurs specifically when E is between V_rest_ and AP threshold, as with E_Cl_ (Staley and Mody, 1992; Wolfart et al., 2005; Meier et al., 2015). There are few but notable differences between the iKA mouse model and the human condition. Human GCs lack the GABA_A_ leak increase and instead show an HS-related enhancement of ZD7288-sensitive, hyperpolarization-activated cation conductance, most likely mediated by HCN1 channels (Stegen et al., 2012). Thus, contrary to prior reports (Stabel et al., 1992), a functional h-current (I_H_) exists in rodent and human GCs (Young et al., 2009; Stegen et al., 2012) and this I_H_, as well as respective HCN1 subunits are enhanced in HS-related TLE (Bender et al., 2003; Stegen et al., 2012). The functional effect of I_H_ in GCs is similar to the GABA_A_ leak because E_H_ is also between V_rest_ and AP threshold, again contributing to enhanced shunting inhibition (Stegen et al., 2012) (see CA Section for more discussion on HCN channels). Another interesting difference between the GCs of TLE patients and iKA mice is that human GCs almost never display a pronounced delay of AP responses as their iKA counterparts (Stegen et al., 2009, 2012; Young et al., 2009). These “ramp” delays of GCs are mediated by *shaker*-related, voltage-gated K (K_v_1) channels containing K_v_1.1, K_v_1.2, or K_v_1.6 subunits, which are sensitive to dendrotoxin and μM concentrations of 4-aminopyridine (4-AP) (Kirchheim et al., 2013). The molecular mechanism for the 3-fold delayed AP responses of iKA GCs is transcriptional upregulation of K_v_1.1 subunits, which, consistent with a homeostatic response, is reversible upon interruption of chronic hyperexcitation (Kirchheim et al., 2013). Without these K_v_1 currents, GCs are much more vulnerable during excitotoxic insults (Kirchheim et al., 2013). The dissimilarities between human and mouse GCs could be due to species differences or (more likely) due to the disease etiology. However, for the present perspective on AED strategies it is notable that in both human and mouse TLE GCs, a depolarizing but shunting conductance is co-upregulated with K_ir_ channels such that V_rest_ is almost unchanged (Stegen et al., 2009, 2012; Young et al., 2009). This downscaling is not only suitable to maintain basic metabolic functions dependent on V_rest_; it is also a native example of static shunt, enforcing a subtractive gain shift of the neuronal input-output curve (Wolfart et al., 2005). A recent network simulation study has demonstrated that the experimentally observed channel scaling of GCs could also restore spatiotemporal pattern separation under epileptic conditions, i.e., maintain the proposed function of the DG network (Yim et al., 2015).

In addition to the discussed leak channel modifications, other epilepsy-related changes occur in excitatory ion channels and ionotropic receptors of DG cell types (Table 1). For example, voltage-gated Ca (Ca_v_) channels of P/Q-type (Ca_v_2.2) were found increased while the L-type subunit Ca_v_1.2 was diminished in the DG molecular layer of TLE patient vs. autopsy samples (Djamshidian et al., 2002). No such changes were observed in the TLE model of ventricular KA injection (Westenbroek et al., 1998). Many immunohistochemistry studies exist on GABA_A_R and GABA_B_R changes during TLE: the GABA_A_R α1-3, β2-3, and γ2 subunits were all found elevated in GCs of TLE patients (Loup et al., 2000) and similar results (except α2) were obtained in the iKA model (Bouilleret et al., 2000; Knuesel et al., 2001). In sPilo, the DG immunostaining of GABA_A_R subunits is heterogeneous (Brooks-Kayal et al., 1998). Some of the confusion, created by various tissue-level studies was clarified by electron microscopy and functional analysis. For example, Sun et al. (2007) revealed that although GABA_A_ α4 subunits are reduced extrasynaptically in GCs of the hKindl model, they are in fact increased in synaptic locations which was interpreted as proepileptic. Similarly, GABA_A_γ2 subunits disappear from synaptic locations, reducing the phasic inhibition in sPilo but they reappear in extrasynaptic locations, apparently replacing lost GABA_A_δ subunits because functionally, tonic inhibition is maintained (Zhang et al., 2007). Measuring surface-coupled protein during sPilo yielded reduction of GABA_A_β2/3γ2 subunits on GC membranes; however, GC recordings revealed that tonic GABA_A_ currents were, if anything, increased (Goodkin et al., 2008). In contrast to GABA_A_Rs, the GABA_B_R1a-b immunosignal of GCs was found reduced in HS-TLE patients (Munoz et al., 2002).

Interneurons of the DG degenerate in the hilus and the molecular layer but somatic inhibition of GCs is apparently spared (Magloczky and Freund, 2005). Here we refrain from reviewing TLE-related interneuron numbers, as respective cell counting studies mostly rely on Ca binding proteins which themselves change in TLE as shown by Magloczky et al. (1997); for review see Magloczky and Freund (2005). With respect to ion channels of interneurons in the hilus, a decrease of voltage-gated Na type 1 (Na_v_1) channels (Qiao et al., 2013) and an increase of Ca_v_1 channels (Xu et al., 2007) had been reported. Interestingly, in basket cell interneurons of the DG, tonic GABA_A_ currents are homeostatically adjusted in sPilo rats (Yu et al., 2013).

Astrocytes may also play an important role in epilepsy, in particular via their (potentially impaired) capacity to buffer extracellular K ions (Bordey and Sontheimer, 1998; Jabs et al., 2008; Boison, 2012). For example, in the DG, less glial K_ir_ current was measured in HS- vs. noHS TLE tissue (Hinterkeuser et al., 2000) and less K_2P_3.1-positive glia was noted in the molecular layer (Kim et al., 2008b). Concerning the Kir channels, it is likely that downregulation of the K_ir_4.1 subunit is responsible (Buono et al., 2004; Heuser et al., 2012). However, in another sPilo study, no changes of hippocampal K_ir_4.1 protein were detected or even upregulation was noted in cortical and subcortical regions (Nagao et al., 2013). In a different model (seizure-sensitive gerbils), upregulation of K_2P_3.1 channels was reported (Kim et al., 2007a). In two human cases, gain of function mutation in the glial K_ir_4.1 channel was associated with infantile epileptic spasms (Sicca et al., 2011).

In summary, pro- and anticonvulsive channel changes have been described in the DG of different TLE models. For noHS models, it is difficult to draw a final conclusion on channel changes in GCs as these are heterogeneous. In TLE tissue with HS, the ion channel expression of GCs is clearly changed to decreased excitability.

## Cornu ammonis

A prominent example of acquired channelopathy is the reduced influence of A-type (rapidly inactivating) K channels in dendrites of CA1 PCs of sPilo rats (Bernard et al., 2004). The respective identification as A-type current was performed in current-clamp experiments via 5 mM 4-AP (Bernard et al., 2004), which blocks delayed rectifier (not rapidly inactivating) K_v_1 channels as well as A-type K_v_4 and K_v_1 channels (Pongs, 1992). In addition, a reduction of K_v_4.2 and K_v_1.4 mRNA was detected via RT-PCR from microdissected CA1 tissue with mixed cell types (Bernard et al., 2004). The conclusion that K_v_4.2 channel downregulation mediated the dendritic channelopathy in sPilo rats (Bernard et al., 2004) was confirmed later by immunocytochemistry, although quantification on the tissue level yielded no difference in the chronic phase (Monaghan et al., 2008). In another sPilo study, K_v_4.2 protein of the CA1 region was increased during the first week after SE but decreased in the chronic phase (Su et al., 2008). In the sKA model, K_v_4.2 mRNA was reported unchanged in CA1 PCs in the first 24 h but appeared upregulated 12 h later (Francis et al., 1997). In the same model, A-currents of CA1 PCs were decreased but their sensitivity to 50 μM 4-AP and the immunohistochemistry suggested that these currents were rather mediated by K_v_1 than K_v_4 channels (Sosanya et al., 2014). In contrast to the sPilo rats, tissue from TLE patients with HS displays similar hippocampal K_v_4.2 mRNA levels compared to patients without HS, and on the protein level K_v_4.2 channels are even elevated, despite the cell loss in the CA1 region (Aronica et al., 2009). In a model of cortical heterotopia, K_v_4.2 channels are also elevated in CA1 PCs (Castro et al., 2001). In other epilepsy models, K_v_4.2 changes were transient (Tsaur et al., 1992) or increased in the chronic phase (Pei et al., 1997) and also K_v_1.2 levels renormalize after the acute phase (Tsaur et al., 1992; Pei et al., 1997). Genetic deletion of K_v_4.2 alone is not sufficient to create epilepsy (Hu et al., 2006) which may be due to compensatory mechanisms.

A second prominent channelopathy scenario for TLE also takes place in the distal dendrites of CA1 PCs: downregulation of HCN channels (Bender and Baram, 2007; Dyhrfjeld-Johnsen et al., 2009; Baruscotti et al., 2010; Noam et al., 2011). Opposite to the described increase in DG GCs, HCN1 channels are decreased in CA1 PCs of both sPilo and sKA rats (Jung et al., 2007; Marcelin et al., 2009; Jung et al., 2011). On the tissue level, upregulation of HCN1 was observed in the CA1-3 regions of sPilo, but it was speculated that this staining could have been due to interneurons (Oh et al., 2012). The HCN-mediated ZD7288-sensitive h-current has a shunting effect not only in DG GCs (Stegen et al., 2012) but also in CA1 PCs (Gasparini and Difrancesco, 1997; Magee, 1999; Berger et al., 2001). Consequently, loss of HCN is usually interpreted as proepileptic (Brewster et al., 2002; Poolos et al., 2002; Jung et al., 2007, 2011; Marcelin et al., 2009). However, there are situations, e.g., in febrile seizure models, where an *increase* of I_H_ in CA1 PCs was interpreted as proepileptic (Chen et al., 2001; Poolos, 2004, 2009; Dyhrfjeld-Johnsen et al., 2008, 2009; Noam et al., 2011). Similar to the A-type channelopathy, the HCN channelopathy of noHS models could so far not be confirmed with human data (Bender et al., 2003). In patients with mild HS, abundant HCN1 protein decorates the distal dendrites of surviving CA1 PCs (Stegen et al., 2012). Similar to K_v_4 channels, HCN1 loss in CA1 PCs alone does not produce epilepsy, but it can enhance the susceptibility for certain seizure induction protocols (Huang et al., 2009; Poolos, 2009; Santoro et al., 2010).

Other ion channel modifications in CA1 PCs include an enhanced Ca_v_ channel function in the sPilo, determined with 0.1 mM nickel (Sanabria et al., 2001; Su et al., 2002). This concentration inhibits Ca_v_2.3 (R-type) and Ca_v_3 (T-type) channels. The Ca_v_3.2 RNA from homogenized tissue was indeed found elevated in sPilo, but only in the acute phase and not in the chronic phase (Becker et al., 2008). Another sPilo study detected no changes or even downregulation of Ca_v_3 RNA, but also only on the tissue level (Graef et al., 2009). Concerning L-type channels (Ca_v_1.2, Ca_v_1.3), P-type (Ca_v_2.1), N-type channels (Ca_v_2.2), and R-type Ca_v_ channels, no immunocytochemistry differences were detected in CA1 PCs of TLE vs. autopsy samples (Djamshidian et al., 2002). Kindled rats displayed an increased window current in CA1 PCs mediated by Na_v_ channels at V_rest_ (Ketelaars et al., 2001). These differences appeared unlikely to arise from Na_v_1.1, Na_v_1.2, and Na_v_1.6 channels, as their immuno signal was not changed under similar conditions (Blumenfeld et al., 2009). In contrast, a lowered amount of Na_v_1.2 RNA was found via *in situ* hybridization in human TLE vs. autopsy samples (Whitaker et al., 2001). Small conductance, Ca-activated K (SK) channels mediating the medium duration afterhyperpolarization (AHP) appeared reduced in CA1 PCs of sPilo rats and this phenomenon corresponded to a reduction of the SK2 RNA amount on the tissue level (Schulz et al., 2012). Another sPilo study found transient changes in SK1 and SK2 protein and permanent downregulation of SK3 via western blotting of homogenized hippocampus (Oliveira et al., 2010). In a maximal electroshock convulsions (MES) model, no changes were detected in K_v_1.1/2/4, K_v_4.2, and K_ir_3.1/2 channels of CA1 PCs (Pei et al., 1997). In seizure-sensitive gerbils, CA1 PCs displayed elevated K_v_3.1b and K_v_3.2 channel immuno signals (Lee et al., 2009).

The PCs of CA2 and CA3 (in particular CA3a/b) are notably less affected by HS-related cell death than CA1 PCs (Wyler et al., 1992; Blümcke et al., 2012). In samples from TLE patients vs. autopsy, CA3 PCs exhibited increased immunoreactivity for Ca_v_2.1 subunits (Djamshidian et al., 2002). In the same work, the Ca_v_1.2 was diminished in CA3 PCs, but enhanced in astrocytes. The Na_v_1.2 RNA signal was reduced (Whitaker et al., 2001) while the Ca_v_2.1 immuno signal was elevated in CA3 PCs of HS-TLE patients vs. autopsy (Djamshidian et al., 2002). In noHS models, the RNA and immuno intensities of Na_v_1.6 channels were found raised in CA3 PCs (Blumenfeld et al., 2009) while those of K_v_4.2 and K_v_1.1 channels were decreased, respectively (Francis et al., 1997; Sosanya et al., 2014). Because chronic Na_v_ upregulation could lead to depolarization block, functional verification is warranted (Auvin et al., 2008; Cestele et al., 2008). The HCN1 RNA was diminished in CA3 PCs of sKA rats, but not after febrile seizures; the HCN2 appeared elevated in both seizure forms, although some of these signals are transient and may be due to interneurons (Brewster et al., 2002). In the MES model, CA cells show little changes in the tested K_v_1, K_v_4, and K_ir_3 channels (Pei et al., 1997) while in an absence epilepsy model CA3 PCs displayed upregulation of some Na_v_ channels (Xu et al., 2013b). With respect to HS-related iKA seizures, CA3a/b PCs appear similar to DG GCs, i.e., high K_ir_2 levels are found in surviving cells (Young et al., 2009). Thus, more functional characterizations of CA3 cell subtypes in relation to different seizure phenotypes are needed. Also, CA interneurons display ion channel changes in TLE. For example, the oriens-lacunosum moleculare interneurons ventral of the HS area in iKA mice possess reduced I_H_ and show increased oscillatory activity in the gamma frequency (Dugladze et al., 2007).

In summary, also in the CA regions, pro- and antiepileptic channel changes have been described in TLE. In comparison with DG GCs, particularly the CA1 PCs stand out with proepileptic changes, as here the two most prominent examples of acquired channelopathy have been described. However, in resected tissue of TLE patients no hyperexcitability was detected in CA1 (Cohen et al., 2002). Thus, the CA1 PC-based acquired channelopathy hypothesis either has to be limited for TLE models without HS or it has to be demonstrated that CA1 PCs in non-sclerotic areas of TLE patients are intrinsically hyperexcitable and/or that surviving hyperexcitable CA1 PCs within the sclerotic hippocampus are connected in a hub-like manner (Morgan and Soltesz, 2008). In its current form, the CA1 PC channelopathy hypothesis collides with the simple principle “dead cells do not seize” (Delorenzo et al., 2005).

## Beyond the hippocampus

Although the entorhinal cortex is a likely source of TLE seizures (Spencer and Spencer, 1994; Spencer, 2002), it received less attention compared to the hippocampus. Nevertheless, there is evidence for ion channel alterations in this region. For example, in layer III PCs of sKA rats, the I_H_ was found decreased in the chronic phase although western blot signals of HCN1 and HCN2 channel subunits were at control level at the same time (Shah et al., 2004). In kindled rats, the neocortical layer II stellate cells display elevated Na_v_ currents and Na_v_1.2/1.6 immunostaining (Hargus et al., 2013). In contrast, a sPilo study found no changes in the intrinsic excitability of layer II PCs and concluded that loss of interneurons was responsible for the elevated perforant path output (Kobayashi et al., 2003). Similarly, K_v_1.1 reduction in cortical interneurons could play a role in some forms of TLE (Li et al., 2012). In frontal lobe epilepsy, layer II/III PCs displayed h-current downregulation (Wierschke et al., 2010). In some forms of cortical epilepsies, the opposite of the above described hippocampal K_v_4.2 channelopathy occurs; here these channels are upregulated suggesting homeostatic adaptation of cortical neurons (Aronica et al., 2009).

Other brain areas connected to the hippocampus also display epilepsy-related ion channel adaptations or pathologies. For example, in the subiculum, increased Ca currents were detected in sPilo (Wellmer et al., 2002) while in kindling models, the amygdala showed elevated levels of KCNQ2 (K_v_7.2) channel protein (Penschuck et al., 2005). Two brain areas rarely implicated in TLE, are the substantia nigra (Depaulis et al., 1994; Ma et al., 2007) and the thalamus (Bertram et al., 2001). One sPilo study found elevated T-type currents in thalamic relay cells and consistent Ca_v_3 channel RNA upregulation on the tissue level (Graef et al., 2009). In contrast, the enhanced K_v_4.2 surface protein expression in ventromedial thalamic relay neurons of sPilo argued for an adaptive response (Smith et al., 2012). We use the thalamus to illustrate that similar ion channels can have opposite functions in different cell types and epilepsies: when sufficiently hyperpolarized, thalamic neurons are capable of rebound burst firing which is mediated by T-type (Ca_v_3) channels and thought to underlie absence epilepsy (Budde et al., 2005; Cope et al., 2009; Kanyshkova et al., 2012) although other mechanisms may also play a role (Crunelli and Leresche, 2002; Strauss et al., 2004; Kole et al., 2007). Therefore, it makes sense that absence epilepsy is treated with Ca_v_3 channel inhibitors, while hyperpolarizing AEDs can even aggravate absence seizures (Rogawski and Löscher, 2004; Powell et al., 2014). Bursting of “epileptic neurons” has long been suspected as the cellular correlate of epileptic seizures in general (Sypert and Ward, 1967). Also for TLE, pathological Ca_v_3-supported burst firing has been suggested as a cellular mechanism for seizures (Yaari and Beck, 2002). However, the same T-type channel responsible for epileptic bursting in thalamic and cortical neurons could actually prevent burst firing in other cell types (Wolfart and Roeper, 2002). Hence, it is important to determine the role of AED-targeted ion channels in a cell type-specific manner, in particular since current AEDs are applied systemically.

## Mechanisms underlying epilepsy-related ion channel alterations

The molecular upstream links of many of the above described ion channel modifications are unclear. For example, the correlation between HS and the leak channel upregulation in DG GCs currently only allows the statement that some part of the sequence, which leads to HS, must also be responsible for the observed changes in channel transcription. One possibility would be that morphological alterations, e.g., related to cytoskeletal changes as they occur during GCD, directly also cause the channel changes (O'Malley and Harvey, 2007). As GCD is caused by reelin deficiency (Haas et al., 2002), reelin is a candidate molecule and indeed it can affect transcription factors (Feng et al., 1999; Chen et al., 2007). However, the hypothesis requires that reelin deficiency alone (without epilepsy) must also trigger the respective channel changes, which does not appear to be the case (Kowalski et al., 2010). Another possible mechanism for transcriptional channelopathies is that the access to neurotrophic factors is interrupted in areas of injury (Waxman, 2001).

We currently favor the hypothesis that the seizures themselves partially cause HS (Mathern et al., 2002) and that the same seizure-induced mechanisms which cause HS, also cause the ion channel changes. The reasoning is as follows. In animal models, SE-related excitotoxic neurodegeneration has two phases: an acute glutamate receptor overstimulation cell swelling phase, and a late Ca-related phase, which gradually fades into the chronic phase of spontaneous seizures, the latter being associated with permanently disturbed intracellular Ca levels (Olney and Sharpe, 1969; Choi, 1992; Magloczky and Freund, 1995; Delorenzo et al., 2005). Also for the iKA model two phases of HS have been described; in the first phase, i.e., hours after KA injection, CA1 PCs and hilar neurons degenerate; in the second phase, about 2 weeks after KA injection, full HS develops with GCD and neurodegeneration affecting CA1, CA3c PCs and eventually also CA3a/b, CA2 PCs, and DG GCs (Bouilleret et al., 1999). While the initial injury has its own channel changes (see below), the second phase of HS-related neurodegeneration in iKA mice correlates well with the described downscaling GC excitability in the same model (Young et al., 2009). Another argument for the hypothesis “chronic seizures cause chronic channel adaptations” is the evidence showing that activity-dependent Ca signals directly couple the degree of excitation to the regulation of gene expression (Marder and Goaillard, 2006). Furthermore, hippocampal seizures are associated with extensive NMDA receptor activation and Ca influx inducing cell stress and neurodegeneration (Isokawa and Levesque, 1991; Magloczky and Freund, 1995; Golowasch et al., 1999; Limbrick et al., 2003; Raza et al., 2004; Ayala and Tapia, 2005; Suzuki et al., 2005). From what we know about the mechanisms of long term synaptic potentiation (LTP), the NMDA-mediated Ca-influx during seizures is likely to activate Ca-dependent kinases (Varga et al., 2004; Nassirpour et al., 2010) and transcription factors, which in turn regulate the transcription of specific ion channel genes (Scharfman, 2002; Fan et al., 2005; Blair et al., 2008; Mucha et al., 2010). This view is also consistent with many homeostasis studies demonstrating how increased neuronal activity can directly influence ion channel expression (Turrigiano et al., 1995; Desai et al., 1999; Van Welie et al., 2004; Misonou et al., 2006). Even the subunit-specific heteromerization can be influenced by seizure-like activity (Zha et al., 2008). Another aspect of epilepsy-related channel regulation is the subject of age. Generally, the immature brain appears more sensitive to seizures than the adult brain (Jensen and Baram, 2000), but chronic epilepsy may develop easier in adult animals (Brooks-Kayal et al., 1998; Zhang et al., 2004; Raol et al., 2006b).

Many mechanistic questions remain. For example: if TLE models without HS show similar elevated input to GCs as HS models (Kobayashi et al., 2003; Kumar and Buckmaster, 2006), why do they not display the same channel changes as HS-TLE models? We would like to know what kind of patterns evoke which type of homeostatic ion channel regulation. There are many molecules modified in HS-related TLE (Becker et al., 2003; Elliott et al., 2003; Lukasiuk and Pitkanen, 2004; Motti et al., 2010; Okamoto et al., 2010). Which of these molecules relates to ion channel transformations? A major question is: if the hypothesis of activity- and Ca-dependent homeostatic channel regulation is true, why are there so few adaptive changes in CA1 PCs (Whitaker et al., 2001; Ge et al., 2011)? One possibility would be that CA PCs have a network task that does not allow such homeostatic adaptations. For example, if activity-dependent LTP strengthens synapses on CA1 PCs, it could be counterproductive if the same activity would weaken synaptic impact. In this context, it makes sense that LTP-inducing mechanisms are accompanied by permissive K_v_ channel internalization (Kim et al., 2007b; Hyun et al., 2013) or HCN1 downregulation (Mcclelland et al., 2011). On the other hand, it is unlikely that LTP of CA1 PCs is a positive feedback mechanism that exists without homeostatic ion channel control (Abbott and Nelson, 2000). Astonishingly, the survival rate of CA1 PC cells is even higher in the noHS TLE models where exactly these detrimental mechanisms have been discovered. If noHS TLE would be an early stage of HS TLE, the LTP-permissive channel regulation of CA1 PCs could be even stronger in HS-related TLE and in fact be responsible for the CA1 PC degeneration. Back to the initial question: CA1 PCs are indeed also capable of homeostatic ion channel regulation counteracting chronic hyperexcitability (Van Welie et al., 2004, 2006; Otto et al., 2006). Thus, similar to the interaction of LTP and synaptic depression, there must be mechanisms to separate homeostatic and LTP-permissive ion channel regulation within the same neuron.

In addition to the control of gene transcription, epilepsy-related changes in ion channel function can be fine-tuned via post-transcriptional mechanisms like splicing and RNA edition, as well as oxidation or phosphorylation. For example, glycine receptors of hippocampi resected from TLE patients show altered RNA editing, which is particularly relevant when combined with abnormal expression of Cl cotransporter 2 (KCC2) and proconvulsive shift of E_Cl_ (Eichler et al., 2008; Meier et al., 2014). Another example is the increased K_v_1.1 RNA editing found in sKA rats (Streit et al., 2011). The intracellular redox state is also known to influence ion channel function via post-translational modulation (Ruppersberg et al., 1991) and redox-sensitivity of A-type channels appears to be modified in DG GCs from sPilo rats (Rüschenschmidt et al., 2006). Hypoxia is another stimulus for adaptive ion channel modification. For example, in CA1 PCs, reduced oxygen levels lead to I_H_ downregulation within less than an hour (Zhang et al., 2006). Seizure-related pH changes can also affect channel function and vice versa (Ziemann et al., 2008). Furthermore, phosphorylation is a cellular mechanism, not only to regulate protein trafficking but also to dynamically control ion channel gating (Levitan, 1994). For example, the above discussed (sKA K_v_4 and sPilo HCN) channelopathies had both been linked to the intracellular phosphorylation status (Lugo et al., 2008; Jung et al., 2010). Finally, reciprocal changes in phosphorylation and methylation of Na_v_ channels had been observed with sKA-related seizures (Baek et al., 2014).

Ion channel function depends on the subcellular location to which the channels are targeted and this targeting can be altered in epilepsy (Chung et al., 2006). One example is the subcellular redistribution of dendritic K_v_4.2 channels in sPilo rats from the inner to the outer molecular layer of the DG (Monaghan et al., 2008). Another example is the disturbed HCN channel trafficking into dendrites of CA1 PCs in epilepsy models (Shin et al., 2008). Furthermore, the axon initial segment and presynaptic terminals have been specifically implicated in TLE (Wimmer et al., 2010; Meier et al., 2014). Thus, ion channels are constantly adapted on pre- and post-translational levels and epilepsy interferes with both levels. Nevertheless, it may be possible to separate the channel changes into those which are part of a cure, i.e., homeostatic in nature, and those which are part of the disease, i.e., either straightforward channelopathy or failure of homeostasis. In any case, AEDs must act on the background of these channel changes. In the next chapter we ask which AEDs have mechanisms of actions comparable to the homeostatic channel regulations observed in neurons during TLE.

## Ion channel-related antiepileptic drug and gene therapy mechanisms

The available AEDs are hypothesized to work via reducing the impact of excitatory ion channels and/or increasing the effect of net inhibitory channels (Löscher et al., 2013). For example, Phenytoin, Carbamazepine, Lamotrigine, Oxcarbazepine, Zonisamide, Rufinamide, Lacosamide, and Eslicarbazepine are thought to act via Na channels, whereas Ethosuximide, Gabapentin, Pregabalin are Ca channel antagonists. Phenobarbital, Primidone, Diazepam, Clonazepam, Clobazam, Progabide, Vigabatrin, and Tiagabin support inhibition via GABA_A_ channels. Other AEDs like Valproate, Felbamate, and Topiramate are effective at multiple of the above targets. There is only one K channel enhancer AED on the market: the recently approved, first-in-class AED Retigabine (Faulkner and Burke, 2013). Thus, current AEDs mainly target excitatory channels while in contrast, the neurons frequently upregulate K channels when faced with epileptic hyperexcitability (Table 1). Despite the dissimilar routes of AED mechanisms and cell type-specific homeostasis, some of the cellular strategies are indeed comparable to (potential) AED mechanisms (Table 2). More than 100 K channel subunits are currently known (Coetzee et al., 1999; Goldstein et al., 2005; Gutman et al., 2005; Kubo et al., 2005; Wei et al., 2005) and interaction of native subunits enlarges the number of K currents that can be considered as potential AED targets in preclinical research considerably. Therefore, we propose that cell type-specific approaches based on endogenous homeostasis mechanisms, could guide target-driven development of AEDs.

Early studies investigated ATP-sensitive K_ir_ (K_ir_6) channel openers such as cromakalim and diazoxide as potential AEDs (Alzheimer and Ten Bruggencate, 1988; Gandolfo et al., 1989a,b). These K_ir_6 channel enhancers were also found protective in anoxia-induced seizures (Mattia et al., 1994; Yamada et al., 2001). However, because K_ir_6 channels are also expressed in the periphery and because the substances were ineffective in standard AED testing models, K_ir_6 channel activators may be of limited utility for epilepsy therapy (Wickenden, 2002; Meldrum and Rogawski, 2007). Systemic administration of SK channel enhancer EBIO reduces seizures in certain seizure models but also produced severe adverse effects (Anderson et al., 2006). Also, Cl channels were considered in the context of TLE (Stogmann et al., 2006; Bertelli et al., 2007; Rinke et al., 2010). Retigabine was initially thought to exert its anticonvulsive action only via GABARs (Rostock et al., 1996; Otto et al., 2002). Later it was shown that Retigabine also activates K_v_7 channels, fortunately those of the brain (K_v_7.2-5) and not those of the heart (K_v_7.1) raising hopes on the new AED class (Main et al., 2000; Rundfeldt and Netzer, 2000; Wickenden et al., 2000; Tatulian et al., 2001; Dost et al., 2004). However, to establish how retigabine and similar related compounds are best used in epilepsy therapy, still has to be determined (Splinter, 2013).

The K_v_1.1 channel subunit has often been implicated in epilepsy (Smart et al., 1998; Wenzel et al., 2007; Robbins and Tempel, 2012). A gene therapy approach showed that viral K_v_1.1 overexpression in neocortical PCs of mice with neocortical epilepsy, effectively reduces the respective seizures (Wykes et al., 2012). A K_v_1.1 reduction in interneurons was also suggested to play a role in some forms of TLE (Li et al., 2012). Interestingly, precisely the K_v_1.1 subunits are enhanced endogenously in DG GCs of iKA mice with severe HS (Kirchheim et al., 2013) but apparently not in TLE patients with HS (Stegen et al., 2009, 2012). Thus, K_v_1.1 enhancement in specific hippocampal neurons could be an excellent antiepileptic and neuroprotective strategy (Kirchheim et al., 2013; Sosanya et al., 2014).

A number of drugs, originally approved for a different action, were later found to enhance K_ir_ channel function; e.g., K_ir_1.1 (Pregabaline) and K_ir_2.3 (Tenidap; Liu et al., 2002; Lee and Liou, 2014). Furthermore, supporting K_ir_3 channel (Kaufmann et al., 2013) and K_ir_2 channel (Xu et al., 2013a) function *in vivo* is effective against seizures of certain epilepsy models (Table 2). Finally, K_2P_2.1 upregulation via adenoviral gene therapy reduces EEG seizures in the sPilo model (Dey et al., 2014). Hence, leak K channels could be an attractive AED target. Again, especially K_ir_2.1-2.4 and K_2P_2.1 leak channels, which are endogenously upregulated in DG GCs during epileptic hyperexcitability, appear to be good candidates (Young et al., 2009).

Although enhancing K channel function is a plausible antiepileptic strategy, there are drawbacks. For example, too much silencing via K channels can also be detrimental (Du et al., 2005; Taverna et al., 2005; Coulson et al., 2008). Furthermore, as discussed for the thalamus, de-inactivation of Na_v_ and Ca_v_ channels necessary for AP activation results in the counterintuitive effect that enhancing K currents can increase seizure susceptibility. One example is the big conductance, Ca-activated K (BK) channel. These channels contribute to the fast AHP, enabling high AP frequencies. Consequently, enhancing BK currents has proepileptic effects (Jin et al., 2000; Brenner et al., 2005; Shruti et al., 2008). If in turn the fast AHP is impaired in interneurons, e.g., via loss of K_v_3.2 channels, seizures become more likely (Lau et al., 2000). Hence, the usability of K channel enhancing AEDs has to be carefully evaluated.

As explained, certain Cl and cation conductances, mediated by GABA_A_Rs and HCN channels, confer shunting inhibition which may also be used as an AED strategy. Indeed, enhancing tonic GABA_A_ currents by overexpression of α5/β3/γ2 and α6/β3/δGABARs, reduced epileptiform activity in hippocampal cell culture and elevation of the δ GABARs *in vivo* lowered cyclothiazide (CTZ)-induced seizures (Sun et al., 2013). Also HCN channels are an AED target which requires further investigation (Poolos et al., 2002; Shah et al., 2013): certain AEDs, initially approved as Na_v_ and Ca_v_ channel blockers, later turned out be enhancers of I_H_ in CA1 PCs as well (Poolos et al., 2002; Surges et al., 2003). However, since one of them (Lamotrigine) was also effective in interneurons (Peng et al., 2010), further experiments must clarify to which extend I_H_ is involved in the mechanisms of action of these AEDs. As explained above, the combination of K_ir_ and HCN/Cl channel upregulation achieves a homeostatic shunt in GCs during TLE (Young et al., 2009; Stegen et al., 2012). It is tempting to speculate that support of such shunting channel combinations indicated by GCs (which could be called “channelacoids”) is a particularly promising AED strategy.

Impaired interneuron activity, e.g., due to Na_v_ channel mutations, often increases seizure susceptibility (Lau et al., 2000; Chen et al., 2002; Ogiwara et al., 2007; Martin et al., 2010; Mashimo et al., 2010; Rossignol et al., 2013; De Kovel et al., 2014; Hedrich et al., 2014). Therefore, it makes sense that supporting GABAergic transmission is a successful AED treatment (see above). However, more cell type-specific data is needed to explain how systemically administered AEDs, which block Na_v_ channels also expressed in interneurons, actually work. Apparently these AEDs preferentially target excitatory neurons (Prakriya and Mennerick, 2000; He et al., 2002; Pothmann et al., 2014). On the other hand, functional enhancement of interneurons can be an effective AED strategy (Jensen et al., 2014). The next question would be how interneurons inhibiting interneurons fit into these scenarios (Kim et al., 2008a). Generally, the specific role of interneuron subtypes in epilepsy is far from clear, in particular when considering that many of the synchronous AP rhythms, generated by interneurons, are suspiciously akin to epileptic seizures (Cobb et al., 1995; Cohen et al., 2002; D'antuono et al., 2004; Vida et al., 2006). Last but not least, it should be kept in mind that all ion channel abnormalities acquired during epilepsy, can affect their sensitivity for AEDs. Indeed, changes in the channel subunit composition are among the mechanisms proposed to underlie acquired pharmacoresistance (Sun et al., 2007; Zhang et al., 2007; Streit et al., 2011). One example would be a decrease of the K_v_7.2/7.3 ratio, as suggested by (Otto et al., 2006), which is expected to increase the Retigabine sensitivity of K_v_7 channels (Schenzer et al., 2005).

In summary, cell type-specific information on epilepsy-related ion channel modifications can explain and support AED strategies. Precisely those inhibitory ion channels which appear to be effective AED targets in preclinical tests are the ones upregulated in DG GCs during TLE. These data indicate that cell-endogenous ion channel homeostasis mechanisms could be used as “channelacoid” archetypes in the search of antiepileptic strategies. In particular, the enhancement of static shunt via combined K/Cl/cation leak channel support appears to be a promising strategy.

### Conflict of interest statement

The authors declare that the research was conducted in the absence of any commercial or financial relationships that could be construed as a potential conflict of interest.
